# Ceramide AD™ Restores Skin Integrity and Function following Exposure to House Dust Mite

**DOI:** 10.3390/ijms24119234

**Published:** 2023-05-25

**Authors:** Hanene Bzioueche, Myriam Tamelghaghet, Bérengère Chignon-Sicard, Noémie Bazile, Pauline Hauchecorne, Maria Barbero Calderón, Pauline Meunier, Stéphane Rocchi, Thierry Passeron, Meri K. Tulic

**Affiliations:** 1 Team 12, Centre Méditerranéen de Médecine Moléculaire (C3M), Université Côte d’Azur, INSERM U1065, 150 Route de Ginestière, CEDEX 3, 06204 Nice, France; hanene.hadhiri-bzioueche@univ-cotedazur.fr (H.B.); stephane.rocchi@univ-cotedazur.fr (S.R.); thierry.passeron@univ-cotedazur.fr (T.P.); 2 Group SVR, 91220 Le Plessis-Pâté, France; myriam.tamelghaghet@svr.com (M.T.); noemie.bazile@svr.com (N.B.); pauline.hauchecorne@svr.com (P.H.); pauline.meunier@svr.com (P.M.); 3 Department of Plastic Surgery, Pasteur Hospital, 06000 Nice, France; docteurchignonsicard@gmail.com; 4 Zurko Research, 28023 Madrid, Spain; maria.barbero@zurkoresearch.com; 5 Department of Dermatology, University Hospital of Nice, Côte d’Azur University, 06001 Nice, France

**Keywords:** atopic dermatitis, Ceramides, E-cadherin, house dust mite, keratins, MMP-9

## Abstract

Ceramides are epidermal lipids important for normal skin barrier function. Reduced Ceramide content is associated with atopic dermatitis (AD). House dust mite (HDM) has been localized in AD skin where it plays an exacerbator role. We set to examine the impact of HDM on skin integrity and the effect of three separate Ceramides (AD™, DS, Y30) on HDM-induced cutaneous damage. The effect was tested in vitro on primary human keratinocytes and ex vivo on skin explants. HDM (100 μg/mL) decreased the expression of adhesion protein E-cadherin, supra-basal (K1, K10) and basal (K5, K14) keratins and increased matrix metallopeptidase (MMP)-9 activity. The presence of Ceramide AD™ in topical cream inhibited HDM-induced E-cadherin and keratin destruction and dampened MMP-9 activity ex vivo which was not seen for the control cream or cream containing DS or Y30 Ceramides. The efficacy of Ceramide AD™ was tested in a clinical setting on moderate to very dry skin (as surrogate for environment-induced skin damage). When applied topically for 21 days, Ceramide AD™ significantly reduced transepidermal water loss (TEWL) in patients with very dry skin compared to their TEWL baseline data. Our study demonstrates Ceramide AD™ cream to be effective in restoring skin homeostasis and barrier function in damaged skin and warrants testing in larger clinical trials for possible treatment of AD and xerosis.

## 1. Introduction

Ceramides are lipids naturally present in the skin that make up ~50% of all lipids in the epidermis (by weight) [1]. They are composed of sphingosine and fatty acids and are known to play an important role in skin elasticity and maintaining the water permeability barrier function of the skin [2]. Ceramides help with the formation of the cutaneous barrier to keep the skin hydrated and are thought to protect the skin from external aggressions and insults such as pollution, toxins, and environmental irritants and allergens. Alterations in Ceramide content in the skin are associated with a number of skin diseases including atopic dermatitis (AD) [3,4]. Supplementation of Ceramides [5] or the use of Ceramide-like creams [6] have been shown to ameliorate dry skin symptoms in the skin of patients with AD, or in patients with contact dermatitis [7], and to assist in the formation of the permeability barrier which in turn can heal damage to the skin barrier [8,9]. Interestingly, some Ceramides have been shown to have inhibitory properties against proteases [10]. Thus, in addition to their emollient properties, they might also help to fight against environmental aggressors. 

One of the well-known environmental irritants is the house dust mite (HDM) [11,12]. HDM is an ubiquitous and perennial allergen and an undisputed trigger of allergic diseases including allergic rhinitis, atopic dermatitis [13], and asthma [14]. HDM contains potent allergens (*Dermatophagoides pteronyssinus* Der p1), proteases and components of both bacterial (lipopolysaccharide, LPS) and fungal (β-D-glucan) cell wall, all of which are known allergic triggers and powerful immunomodulators proven to disrupt respiratory epithelia and tight junctions. We have previously demonstrated HDM to be also present in the human gastrointestinal tract where it can disrupt tight junctions between gut epithelial cells and exacerbate gut dysfunction in susceptible individuals [15]. In addition to their important role in respiratory and gastrointestinal diseases, the concept of mites inducing pathogenesis in human skin is well known for atopic dermatitis as well as acne *vulgaris* and *rosacea* [16]. Moreover, in addition to allergenic properties, there is evidence to suggest that the potent protease activity of mites can disrupt the skin epithelial barrier, promoting trans-epidermal water loss and facilitating the penetration of allergens directly into the skin [17]. We have recently shown that in human skin, HDM increased keratinocyte production of vitiligo-associated cytokines and chemokines [18]. This was associated with increased in situ MMP-9 activity, reduced cutaneous expression of adherent protein E-cadherin, increased soluble E-cadherin in culture supernatant and significantly increased number of supra-basal melanocytes in the skin. This effect was dose-dependent and driven by HDM-containing cysteine protease *Der p1* and MMP-9 [18]. 

Dry skin and an impaired barrier function are the hallmarks of AD, and protease activity of environmental triggers such as airborne mites may play a previously underestimated role in the pathogenesis of AD [19]. There is mounting evidence that environmental contribution to skin diseases is an important factor to consider [20], and epidermal proteins affected by mites are not fully characterized, although mites have been detected in naturally occurring lesions of atopic dermatitis [21]. Effective compounds to fight against these aggressors are currently lacking. Here, we assessed the effect of three separate Ceramide formulations, (1) Ceramide AD™ (Ceramosides™ HP), (2) *Dihydrosphingosine* (DS) *Phytoshingosine*, and (3) DS-Ceramide Y30, in their ability to restore or abolish the HDM-induced damage and restore skin barrier dysfunction. Initial screening tests were performed in vitro in primary human keratinocytes on separate Ceramides, followed by ex vivo studies in human skin biopsies with the promising candidate. Finally, the effective Ceramide AD™ was tested in a clinical setting, measuring its ability to reduce skin water loss and restore normal skin function in dry skin.

## 2. Results

### 2.1. HDM Selectively Decreases E-Cadherin Expression in Primary Human Keratinocytes

**Figure 1:**
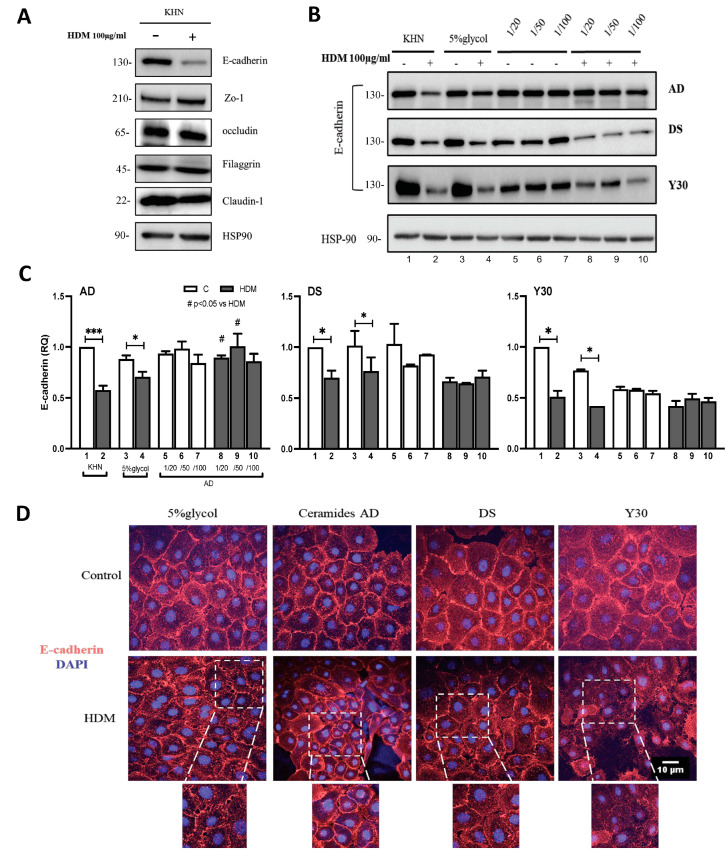
Effect of HDM on expression of adherent and tight junctions between keratinocytes and their modification by Ceramides. (**A**) Primary human keratinocytes (KHN) were stimulated with HDM 100 μg/mL for 24 h and expression of adherent and tight junction proteins assessed by Western blot (WB) analysis. HSP90 was used as a loading control. (**B**) E-cadherin expression in the same cells stimulated with HDM in the presence or absence of Ceramide AD™, DS Phytoshingosine or DS Ceramide Y30 at 1/20, 1/50 or 1/100 dilution dissolved in 5% glycol (vehicle control). (**C**) WB gels were semi-quantitated by densitometric analysis using ImageJ software (version 2.1) from at least 3 separate experiments and normalized to HKG (HSP90) relative quantity (RQ) (*n* = 3). Numbers 1–10 denote corresponding lanes in (**B**). (**D**) Immunofluorescence staining of KHN stimulated with (bottom panels) or without (top panels) HDM 100 μg/mL in the presence of separate Ceramides whereby E-cadherin-positive cells are shown as red and DAPI-positive cells are shown as blue. * *p* < 0.05, *** *p* < 0.001 vs. respective controls and # *p* < 0.05 vs. HDM.

To look at the effect of HDM on adherent and tight-junction proteins between skin cells, we incubated primary human keratinocytes in vitro with HDM 100 μg/mL for 24 h and examined their expression of E-cadherin, Zona Occludin (Zo)-1, occludin, Filaggrin and Claudin-1 by Western blot analysis. Our results showed HDM to selectively decrease the expression of E-cadherin (transmembrane protein that mediates cell–cell adhesion) but not the other tight-junction proteins (Zo-1, occludin or Claudin-1) or filaggrin between human keratinocytes (Figure 1A). HSP90 was used as a house keeping gene (HKG) and the blot is a representative of three separate blots.

### 2.2. Ceramide AD™ but Not DS or Y30 Restored HDM-Induced Destruction of E-Cadherin

We then examined the ability of three different Ceramides: Ceramide AD™, Ceramosides™ HP or DS-Ceramide Y30 (SVR, France) to impact HDM-induced changes in keratinocytes in vitro. We tested three dilutions of each compound (1/20, 1/50 and 1/100) and examined their effect on E-cadherin protein expression 24 h after stimulation with 100 μg/mL HDM. Our results confirmed decreased a expression of E-cadherin in keratinocytes stimulated with HDM (and its vehicle control 5% glycol) and this decrease was inhibited only in the presence of Ceramide AD™ but not in the presence of Ceramosides™ HP- or DS-Ceramide Y30-treated cells (Figure 1B). These responses were semi-quantitated from original gels from three independent experiments (Figure 1C). These results were confirmed ex vivo in human skin explants using immunofluorescence staining, showing Ceramide AD™-treated keratinocytes to protect against HDM-induced destruction of E-cadherin (second column, bottom) and these responses to be similar to E-cadherin expression seen in the vehicle control condition (5% glycol) without HDM exposure (top left) (Figure 1D). Keratinocytes treated with DS or Y30 Ceramides failed to restore E-cadherin destruction following HDM exposure (Column 3 and 4) (Figure 1D). In the zoom area of interest, we can see well-defined keratinocyte border outlines (Column 2) suggesting cellular integrity, and the lack of these adhesion borders is still apparent with HDM in the presence of DS or Y30 treatment (Column 3 and 4 are representatives of three separate experiments). Together, these results demonstrate the efficacy of Ceramide AD™ but not Ceramides DS or Ceramides Y30, in restoring HDM-induced E-cadherin damage. For this reason, the rest of the study was focused on Ceramide AD™.

### 2.3. Ceramide AD™ Increases Basal Keratin Content

**Figure 2:**
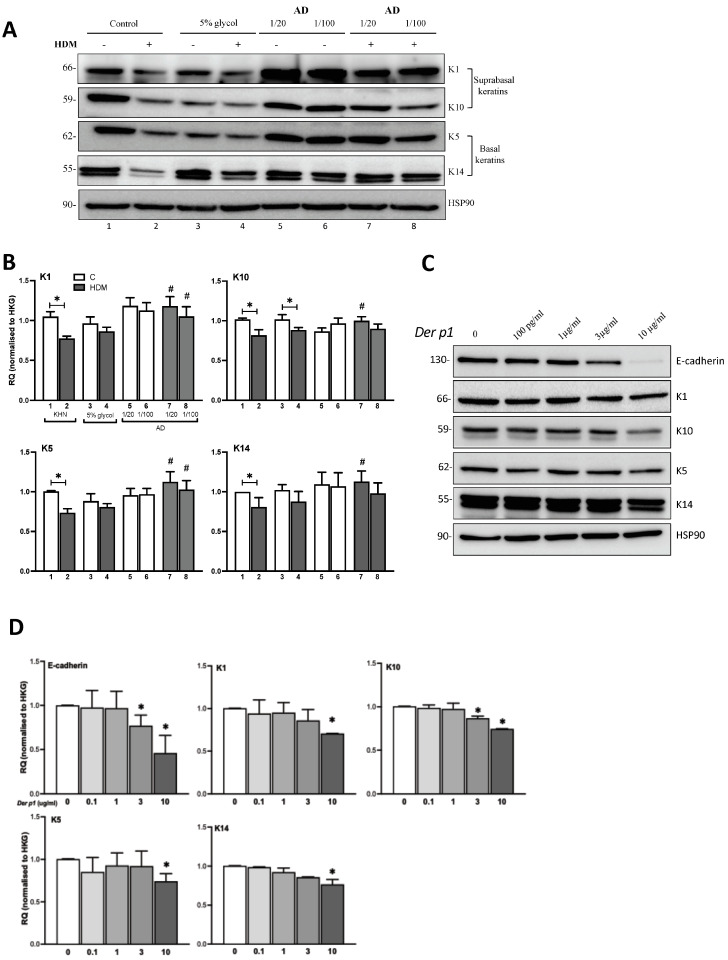
Effect of HDM and *Der p1* on keratin expression and modification by Ceramides. (**A**) Supra-basal keratin (K1, K10) and basal keratin (K5, K14) expression in primary human keratinocytes (KHN) stimulated with HDM 100 μg/mL for 24 h in the presence or absence of Ceramide AD™ at a 1/20 or 1/100 dilution in 5% glycol. (**B**) WB was semi-quantitated by densitometry and results expressed as RQ normalized to HKG (*n* = 5–7). Numbers 1–8 denote corresponding lanes in (**A**). (**C**) E-cadherin expression and expression of all keratins was assessed in keratinocytes stimulated for 24 h with increasing concentrations of *Der p1* (100 pg/mL–10 μg/mL) and semi-quantitated by densitometry assessment (**D**) All semi-quantification of the WB was performed by ImageJ software (version 2.1) and results were represented as RQ normalized to HKG HSP90 (*n* = 3). * *p* < 0.05 vs. respective controls and # *p* < 0.05 vs. HDM.

To check if Ceramide AD™ can reduce HDM-induced changes in keratinocyte integrity, we examined the expression of supra-basal keratins K1, K10 (specifically expressed in the spinous and granular layers of the epidermis) and basal keratins K5, K14 (specifically expressed in the basal layers of the epidermis). Our WB results demonstrate that HDM was able to decrease the expression of all keratins tested, which was restored following addition of Ceramide AD™ (Figure 2A). These results were semi-quantitated from the original gels of three separate experiments and confirmed significant reduction in all keratins tested following HDM exposure (*p* < 0.05) (Figure 2B) and Ceramide AD™ to restore completely HDM-induced keratin destruction, even when used at a 1/100 dilution for K1 and K5 (Figure 2B). To examine whether the effects seen with HDM were driven by *Der p1*, its major enzyme with strong protease activity, keratinocytes were stimulated in vitro with increasing doses of *Der p1* (100 pg/mL to 10 mg/mL) and keratin expression examined at 24 h. Our results showed *Der p1* to induce a decrease in the expression of E-cadherin and all keratins tested (Figure 2C). This decrease was dose dependent as shown by the quantification plots of separate blots (Figure 2D). It is noteworthy that the quantity of *Der p1* in our HDM extract corresponds to ~3 µg/mL and is hence comparable to the effects seen. Together, our results suggest that HDM can disrupt the integrity between keratinocytes in vitro by inhibiting their expression of adherent junctions (E-cadherin) and structural skin proteins (keratins) and that Ceramide AD™ is able to restore these extracellular changes when present in a culture with HDM (or its major protease *Der p1*).

### 2.4. Impact of HDM and Treatment with Ceramide AD™ on Ex Vivo Skin Model

**Figure 3:**
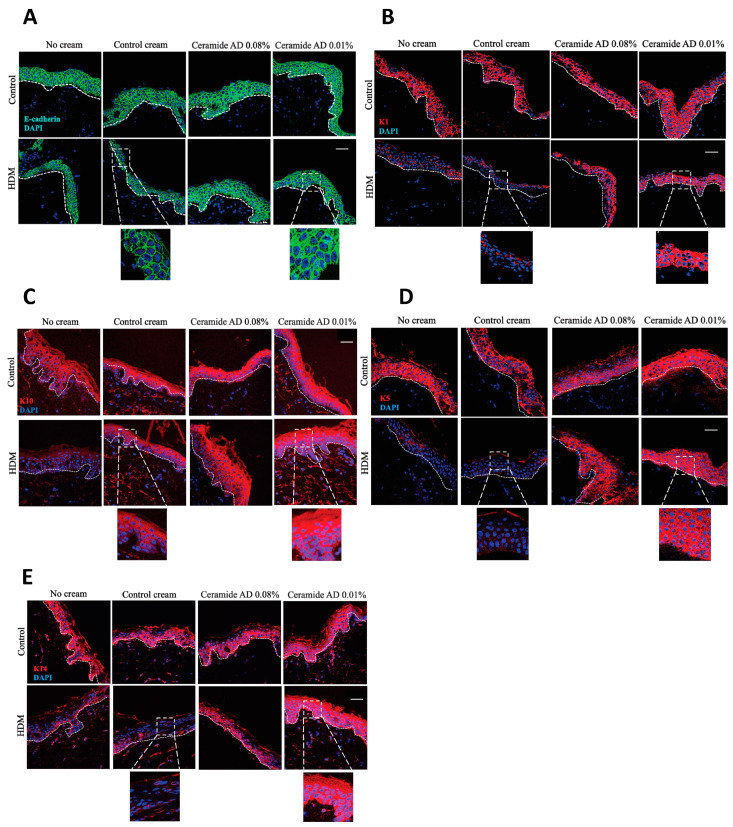
Effect of Ceramide AD™ on HDM-induced changes in the human skin. Immunofluorescence staining of (**A**) E-cadherin (green), (**B**) K1 (red), (**C**) K10 (red), (**D**) K5 (red) and (**E**) K14 (red) in human skin biopsies exposed to HDM 100 μg/mL for 30 min before application of Ceramide AD™ cream at 0.08% or 0.1%. Control conditions tested were without cream or the use of Control cream which did not contain Ceramide AD™. DAPI-positive cells are shown as blue. Scale bar = 10 μm.

Given these encouraging results in vitro, Ceramide AD™ was integrated into a cream at two different concentrations (0.08% and 0.1%) to be tested ex-vivo on human skin biopsies (*n* = 3). The biopsies were oriented with epithelium exposed to the air, forming an ai–-liquid interface to mimic the in vivo situation. They were maintained in skin long-term culture medium (Biopredic International, Saint-Grégoire, France) at 37 °C in a 5% CO_2_ atmosphere. Biopsies were stimulated with HDM 100 μg/mL (Stallergenes, Greer, Lenoir, NC, USA) for 30 min before the cream was directly brushed onto the skin and left for 24 h. The immunofluorescence images of normal skin exposed to HDM with no cream (bottom left panel for each target) demonstrate HDM-induced destruction of E-cadherin (Figure 3A), K1 (Figure 3B), K10 (Figure 3C), K5 (Figure 3D) and K14 (Figure 3E) proteins between skin cells. The Ceramide AD™ cream inhibited the HDM-induced E-cadherin and keratin destruction and restored cellular adhesion dose-dependently for all markers tested, which was not seen for the control cream (Figure 3A–E). In the control, non-HDM-exposed skin (top line in all images), Ceramide AD™ also improved baseline cellular integrity, particularly for K10 (Figure 3C) and K5 (Figure 3D) at 0.1%. The zoomed images (×40) are showing the level of staining in the skin treated with the control cream (second column) or the Ceramide AD™-containing cream (third and fourth column) demonstrating, in all cases, improved cell-to-cell adhesion between cells as shown by increased staining intensity and more defined cellular junctions.

### 2.5. Effect of Ceramide AD™ on MMP-9 Activity

**Figure 4:**
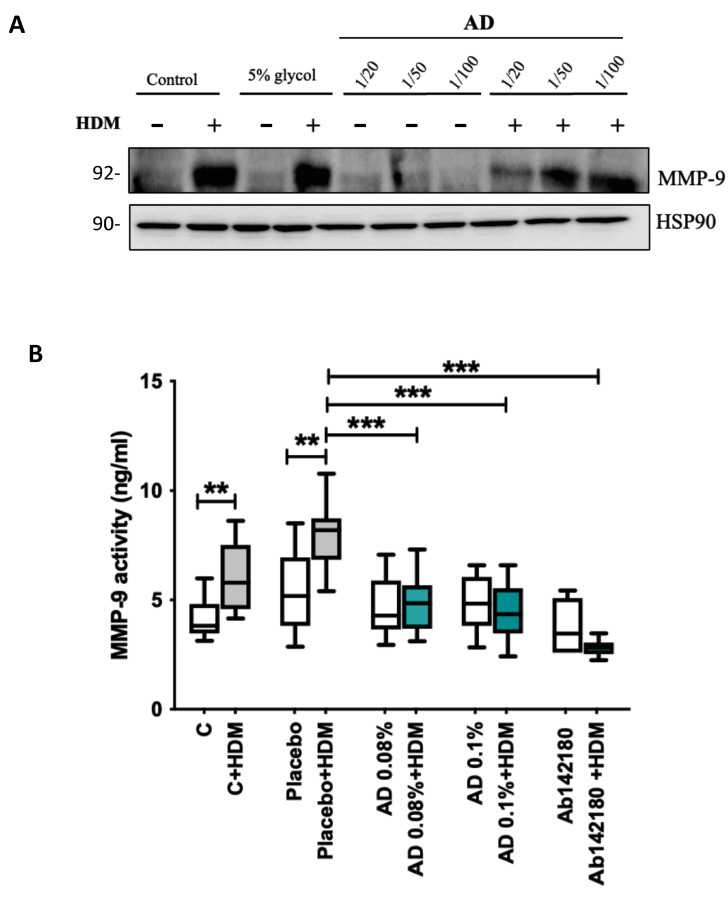
Effect of Ceramide AD™ on HDM-induced MMP-9 activity. (**A**) Expression of the active form of MMP-9 in primary human keratinocytes (KHN) stimulated with HDM 100 μg/mL for 24 h in the presence or absence of Ceramide AD™ at 1/20, 1/50 or 1/100 dilution dissolved in 5% glycol. WB is a representative gel of 3 separate experiments. (**B**) MMP-9 activity in the supernatant was measured by ELISA following HDM stimulation of human skin biopsies with (green) or without (grey) cream containing Ceramide AD™ at 0.08% or 0.1% or using MMP-9 inhibitor Ab142180. Placebo (or vehicle control) was the same cream but did not contain Ceramide AD™ component (*n* = 3 separate experiments). Differences between groups were tested using Wilcoxon Signed Rank Test. ** *p* < 0.01 and *** *p* < 0.001.

First, we checked the effect of Ceramide AD™ on the expression of the active form of MMP-9 in the lysates of KHN 24 h after in vitro stimulation with 100 μg/mL HDM. The results showed HDM increased the expression of the active form of MMP-9 in the lysate of both non-treated controls and vehicle (5% glycol)-treated KHN cells (Figure 4A). KHN treated with Ceramide AD™ inhibited the HDM-induced MMP-9 activity dose-dependently. Ceramide AD™ alone (even at the highest dilution 1/10) had no effect on MMP-9 activity (*n* = 3). These results were confirmed ex vivo in human skin biopsies whereby HDM significantly increased MMP-9 activity in cultured explant supernatants compared to non-treated controls (*n* = 3, *p* < 0.01). HDM similarly increased MMP-9 activity in explants pre-treated with HDM before application of placebo (vehicle)-containing cream on the skin compared to skin biopsies treated with placebo cream alone (*n* = 3, *p* < 0.01) (Figure 4B). There was no difference between unstimulated or placebo-treated controls. Skin treatment with AD 0.08% or AD 0.1% significantly inhibited the HDM-induced MMP-9 increase seen with placebo cream (*n* = 3, *p* < 0.001) which was also seen with a selective MMP-9 inhibitor, Ab142180 (*n* = 3, *p* < 0.001). Treatment with AD alone or Ab142180 alone had no effect on MMP-9 activity (Figure 4B).

### 2.6. In Vivo Effect of Ceramide AD™ on Trans-Epidermal Water Loss (TEWL)

**Figure 5:**
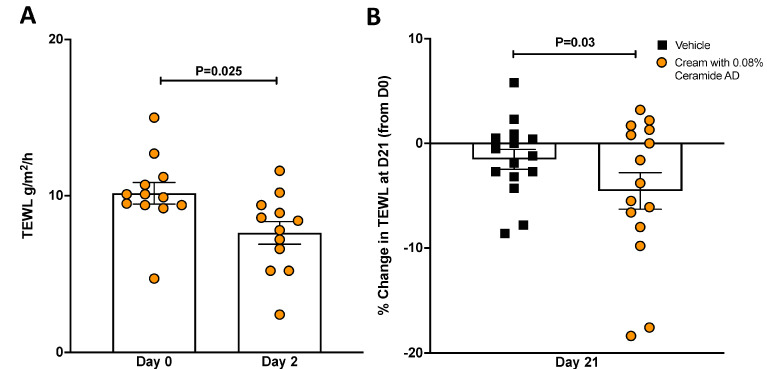
Effect of Ceramide AD™ on Trans-Epidermal Water Loss. Trans-epidermal water loss (TEWL) was measured in 12 patients with (**A**) moderately dry skin (TEWL > 5 g/m^2^/h) (*n* = 12) at baseline (Day 0) and 2 days (Day 2) after daily topical application of the cream. Change in skin function (TEWL) was also measured (**B**) in patients with very dry skin (TEWL > 15 g/m^2^/h), 21 days after daily application of cream on patient’s forearms containing 0.08% Ceramide AD™. The results are represented as change in TEWL from baseline to day 21, in vehicle (black, *n* = 15) and Ceramide AD™-containing cream (orange, *n* = 15), with negative values indicating reduced TEWL and hence improved skin function. Differences between groups were assessed using Wilcoxon Signed Rank Test.

Given the encouraging in vitro and ex vivo results with Ceramide AD™ in repairing skin damage, two clinical studies were set up to assess the effect of Ceramide AD™ (in a cream formulation) on TEWL on damaged human skin (as a surrogate for environment induced HDM damage). In the first study, 12 subjects with moderately dry skin (TEWL > 5 g/m^2^/h) were recruited. After 2 days of product application daily on their forearms, our results showed that TEWL decreased by 25% on average in the treated area in relation to baseline, and this difference was statistically significant (*p* = 0.025) (Figure 5A). In the second study, in 15 patients with very dry skin (TEWL > 15 g/m^2^/h), this effect remained significant even after 21 consecutive days of application with a cream containing 0.08% Ceramide AD™ when compared to patients’ own baseline TEWL values (*p* = 0.03) (Figure 5B).

## 3. Discussion

The impact of HDM on AD is well recognized but mostly as a potential allergen [13]. Although the protease activity of HDM leading to epithelial tight junction alteration has been showen in the lungs [22] and in the gut [15], this was not assessed before in AD, although the skin is widely exposed to HDM. The barrier dysfunction of lung, gut and skin by environmental proteases plays a key role in the pathogenesis of allergic diseases [23]. Previous studies have suggested that the potent protease activity of mites can disrupt the skin epithelial barrier, promoting trans-epidermal water loss and facilitating the penetration of allergens directly into the skin [24,25,26,27]. Here, we demonstrated that HDM can alter epidermal junctions holding keratinocytes and melanocytes by acting selectively on adherent (E-cadherin) and non-tight junctions (Zo-1, occludin, Filaggrin or Claudin-1). We also showed that HDM alters the expression of keratins, K1, K10, K5 and K14, demonstrating its impact on not only the supra-basal mature and differentiated keratins, but also keratins expressed in the basal layers of the epidermis. These results suggest that HDM is capable of effecting both differentiated and non-differentiated keratinocytes. Moreover, we demonstrate the effectiveness of Ceramide AD™ in restoring HDM-induced damages in the skin by acting on E-cadherin and keratin expression, inhibition of cutaneous MMP-9 activation and improvement of skin water loss function. 

Dry skin and an impaired barrier function are the hallmarks of AD. The proteolytic activity of HDM on E-cadherin and on keratin could promote trans-epidermal water loss and facilitate the penetration of allergens directly into the skin. One of the limitations of this study is that we did not directly test the effect of Ceramide AD™ on TEWL in HDM-exposed skin. One of the reasons is that measuring skin HDM content is not reliable as the mites are often buried deep within the hair follicles and therefore the patch test underestimates the real cutaneous HDM content. For this reason, the levels of *Der p1* allergens in reservoir dust collected from beddings with a vacuum cleaner have traditionally been used as an index of environmental exposure to HDM [28]. Using patients with very dry skin as a surrogate of environmentally induced skin damage was a good option as HDM-induced barrier dysfunction is known to induce an inflammatory environment and skin dryness [29].

Ceramides have demonstrated anti-proteolytic activities [30,31]. We show here that not all the Ceramides share the same efficacy in their ability to protect against environmental HDM. In our study, only Ceramide AD™ (and not the Ceramosides HP or DS-Ceramides Y30) was able to effectively protect from HDM-induced alteration in E-cadherin and keratins. Interestingly, Ceramide AD™ not only prevented their decrease after HDM exposure, but also increased basal expression of E-cadherin and keratins, suggesting that they not only have anti-proteolytic properties, but they also can directly stimulate keratin production in the skin. In addition, we demonstrated that Ceramide AD™ can reduce MMP-9 activity secreted in the supernatant following HDM exposure, similar to the levels seen when we used a potent and selective MMP-9 inhibitor, Ab142180. These results were observed not only in vitro in primary keratinocytes in culture but also ex vivo in human skin biopsies. The effectiveness of Ceramide AD™ was translated in a clinical setting showing a significant improvement in TEWL in volunteers with very dry skin; however, this remains to be formally tested directly on HDM-exposed skin. 

In conclusion, here, we provide evidence of effectiveness of Ceramide AD™ on repair of HDM-damaged skin. Our results show it to be effective in restoring E-cadherin and keratin expression in the epidermis, to effectively reduce expression and activity of potent MMP-9 protease induced by HDM, and to improve skin barrier function in dry skin. These novel findings demonstrate the role of HDM in environment-induced skin damage and emphasize the usefulness of some specific types of Ceramides for restoring skin homeostasis. Those results are particularly important in AD as HDM is a known trigger of disease exacerbation. Prospective randomized large clinical trials further assessing the efficacy of Ceramide AD™ in xerosis and in AD patients are now warranted.

## 4. Materials and Methods

### 4.1. Cell Culture

Primary human keratinocytes were isolated from the foreskin of healthy children and cultivated in keratinocyte serum-free medium supplemented with bovine pituitary extract (25 μg/mL) and epidermal growth factor (0.25 ng/mL) (Promo cell, Heidelberg, Germany). Cells were maintained in a humidified atmosphere with 5% CO_2_ at 37 °C. For the experiments, keratinocytes were stimulated with different concentrations of AD, Y30 and DS Ceramide powder (SVR, Paris, France) diluted in water with 5% glycol at a final percentage of 0.00875%, 0.0035%, and 0.00175%, respectively. Keratinocytes were stimulated with 100 µg/mL of House Dust Mite (HDM) in the presence or absence of Ceramides, and results were examined at 24 h (these doses were used in similar in vitro studies in keratinocytes [32] and concentrations 100-fold lower than those used in animal studies where HDM was directly applied to the skin [33]). 

### 4.2. Western Blot Analysis

Keratinocytes were lysed in RIPA (Thermofisher, Paris, France) cell lysis buffer supplemented with protease inhibitors (Roche, Meylan, France). Protein concentrations were measured using a BCA Protein Assay Kit (Bio-Rad, Marnes-la-Coquette, France). Protein lysates (30 μg) were separated by 10% SDS-PAGE gel electrophoresis and transferred to a PVDF membrane (Millipore, Molsheim, France). E-cadherin, Zo-1, occludin, Filaggrin, claudin-1, Keratin 1, Keratin 10, Keratin 5, Keratin 14, MMP-9 and HSP90 were detected using mouse anti-E-cadherin antibody (1/1000, Becton Dickinson, Franklin Lakes, NJ, USA), rabbit anti-Zo-1 (1/1000, Invitrogen, Waltham, MA, USA), rabbit anti-occludin (1/1000, Invitrogen), mouse anti-Filaggrin (Santa Cruz, Dallas, TX, USA), mouse anti-claudin-1 (Santa Cruz), rabbit anti-Keratin 1 (1/1000, Abcam, Cambridge, UK), rabbit anti-Keratin 10 (1/1000, Abcam), rabbit anti-Keratin 5 (1/1000, Cell Signaling, Danvers, MA, USA), rabbit anti-Keratin 14 (1/1000, Novus, Charles, MO, USA), mouse anti-MMP9 E-11 (1/1000, Santa Cruz), mouse anti–HSP90 antibody (1/1000, Santa Cruz), peroxidase-conjugated goat anti-mouse antibody (1/2000, Dako, Santa Clara, CA, USA) and peroxidase-conjugated goat anti-rabbit antibody (1/2000, Dako). Detection was carried out using the ECL detection system (Bio-Rad) and a chemiluminescent image analyzer (LAS-3000, Fujifilm, Tokyo, Japan). Results were normalized to HSP90 and semi-quantitated by densitometric analysis using ImageJ from at least three separate experiments.

### 4.3. Ex Vivo Skin Culture 

Skin from plastic surgery was used for the development of the ex vivo skin culture model. After subcutaneous fat was removed, we obtained 4 mm biopsies which were rapidly placed into a 0.4 μm Transwell chamber (Corning, Somerville, MA, USA) and maintained under semi-liquid culture conditions in skin long-term culture medium (Biopredic, Saint-Grégoire, France) at 37 °C in a 5% CO_2_ atmosphere. The biopsies were treated with two different concentrations of Ceramide AD™ cream (0.08% and 0.1%) and with a control cream (cream without Ceramide AD™) in the presence or absence of HDM 100 µg/mL (Stallergenes Greer, Lenoir, NC, USA) and with or without MMP-9 inhibitor Ab142180 (Abcam). HDM was administered on top of the skin for 30 min before the cream was brushed directly onto the skin. Twenty-four hours later, the biopsy was frozen in an OCT medium (VWR, Rosny-sous-Bois, France) for subsequent immunofluorescence staining on 7 μm tissue sections and supernatants collected for measurement of MMP-9 activity by ELISA (R&D Systems).

### 4.4. Immunofluorescence Staining

Section slides were fixed with 4% paraformaldehyde (Merck, Martillac, France), permeabilized with Tris-buffered saline (PBS) (Fisher Scientific, Illkirch-Graffenstaden, France) in the presence of 0.3% triton (Euromedex, Souffelweyersheim, France) and blocked for 1 h with PBS 5% BSA (Euromedex, France) plus 10% goat serum (Millipore, France). Sections were then incubated overnight at 4 °C with mouse anti-E-cadherin antibody (1/200, Becton Dickinson), rabbit anti-Keratin 1 (1/200, Abcam), rabbit anti-Keratin 10 (1/200, Abcam), rabbit anti-Keratin 5 (1/200, Abcam), and rabbit anti-Keratin 14 (1/200, Novus). After three PBS washes, the skin was incubated with secondary antibodies, Alexa 488 goat anti-mouse IgG (H + L) and Alexa 594 goat anti-rabbit IgG (H + L), respectively. After subsequent washing, the sections were mounted with Prolong Gold antifade reagent containing DAPI (Thermofisher Scientific, Illkirch-Graffenstaden, France), and images were acquired using Nikon confocal A1R (Nikon, Tokyo, Japan).

### 4.5. Clinical Studies

To determine the efficacy of the Ceramide AD™ in a clinical setting, we recruited 13 healthy subjects (average age 34, range 26–46) with moderately dry skin (trans-epidermal water loss, TEWL > 5 g/m^2^/h) and separate 12 healthy subjects with very dry skin (TEWL > 15 g/m^2^/h) (average age 36, range 28–44). Before inclusion, subjects were first interviewed by a health professional to establish their medical status and suitability for inclusion into the study. All subjects had no known pathologies, previous skin reactions or product intolerance, and all subjects signed an informed consent form before their inclusion. A cream with 0.08% Ceramide AD™ were applied to the forearm once a day for 2 or 21 consecutive days (same site was used in all patients). Placebo (vehicle)-containing cream was applied to the other forearm for the same duration. Subjects were asked not to use any other product during the study. The main objective was to test the TEWL efficacy between the test and the placebo cream by assessing the skin capacitance with Tewameter^®^ TM 300 (Courage + Khazaka electronic GmbH, Köln, Germany) before (baseline) and after (at day 2 or 21) daily application of the creams. Tewameter calculates the density of the evaporating gradient of the skin by measuring skin temperature and humidity (the lower the TEWL, the healthier the skin). During these measurements, the subjects stayed 20–30 min in a climatized room with a temperature of 20 °C ± 2 °C and a relative humidity of 40–60%. Since Ceramides are considered as cosmetic compounds and because all our evaluations were non-invasive, according to the French regulation, we did not require approval from an institutional review board. The study was conducted in accordance with The Code of Ethics of the World Medical Association (Declaration of Helsinki). Wilcoxon signed rank test was used to evaluate the efficacy of the treatment with time. The effect of the treatment on the biometric measurements was interpreted comparing each evaluation time in relation to baseline. A significance value of 0.05 was established (95% confidence interval).

## Data Availability

Data available on request from corresponding author.
